# Influence of the Acoustic Environment in Hospital Wards on Patient Physiological and Psychological Indices

**DOI:** 10.3389/fpsyg.2020.01600

**Published:** 2020-07-21

**Authors:** Tianfu Zhou, Yue Wu, Qi Meng, Jian Kang

**Affiliations:** ^1^Department of Architecture, Shanghai Academy of Fine Arts, Shanghai University, Shanghai, China; ^2^Key Laboratory of Cold Region Urban and Rural Human Settlement Environment Science and Technology, Ministry of Industry and Information Technology, School of Architecture, Harbin Institute of Technology, Harbin, China; ^3^UCL Institute for Environmental Design and Engineering, The Bartlett, University College London, London, United Kingdom

**Keywords:** perceptual attributes, acoustic environment, patient feelings, heating region, hospital wards

## Abstract

Patients in general wards are often exposed to excessive levels of noise and activity, and high levels of noise have been associated with depression and anxiety. Previous studies have found that an appropriate acoustic environment is beneficial to the patient’s therapeutic and treatment process; however, the soundscape is rarely intentionally designed or operated to improve patient recovery, especially for psychological rehabilitation. To gain the most accurate, and least variable, estimate of acoustic environmental stimuli/properties, virtual reality (VR) technology should be used to ensure that other environmental factors are stable and uniform in order to reduce the stimulation of other environmental factors. Therefore, this study aims to discuss the influence of the acoustic environment on patient physiological/psychological indicators and the mechanism of the effect on recovery using VR technology. A digital three-dimensional (3D) model of a hospital room was constructed, and experimental subjects wore VR glasses to visualize a real ward scene. Four typical sound categories were selected to analyze the effect of the acoustic environment on recovery; physiological indicators were monitored, and psychological factors were subjectively evaluated. The results show that music plays an important role in reducing stress as it can aid in a patient’s physiological (skin conduction levels) and psychological stress recovery. Furthermore, mechanical and anthropogenic sounds exert negative effects on a patient’s stress recovery. However, the effect is only limited to psychological stress indicators. The interaction effects of demographic characteristics and the acoustic environment are not significant, and future studies could consider the social–economic characteristics of patients. Based on these findings, we provide evidence that indicates that a hospital’s acoustic environment is an important influencing factor on the stress recovery of patients and can serve as a reference for healthcare architects and policy makers.

## Introduction

The indoor environment as a service carrier is most directly influenced by mental feelings, which are linked to patient comfort and mood. Patients stay in the ward almost all day, highlighting the great importance of providing comfortable conditions in hospital buildings. The comfort environment is considered the most important factor influencing patient feelings (Buckles, 1990). Researchers have measured the noise levels or studied the sound source of various healthcare environments, such as critical care wards (Xie et al., 2013), intensive care units (Xie et al., 2009), and entrance halls (Qin et al., 2011). As shown in previous studies, the noise levels measured in the wards frequently exceed the World Health Organization guideline values (45 dBA) by more than 20 dBA (Berglund et al., 1999; MacKenzie and Galbrun, 2007). Noise is a major public health issue, and noise annoyance is the most common and direct response among people exposed to environmental noise. Noise has been identified as a major stressor in hospitals (Farrehi et al., 2016) and will influence an individual’s physical and mental health.

### Literature Review

The documented association with several diseases and the growing number of exposed persons worldwide (Recio et al., 2016) indicate negative emotional and attitudinal reactions to noise (Okokon et al., 2015). Exposure to noise may interfere with daily activities, feelings, thoughts, rest, or sleep and may be accompanied by negative emotional reactions, such as irritability, distress, exhaustion, and other stress-related symptoms (Beutel et al., 2016). The impacts of stressors on health depend on the complex interactions between stressors and individual coping strategies, which are developed through previous experience, psychology, biology, social factors, competitive stressors, and personality (Jensen et al., 2018). Noise-related health problems are growing, and more severe effects related to cardiovascular morbidity and mortality have been proposed (Belojevic et al., 2011). Studies have found that an increase in daily noise levels of 1 dB(1) resulted in a 6.6% increase in the risk of death in the elderly (Tobías et al., 2015) and have observed a significant increase in blood pressure of 2–4 mmHg after 10 min of high-level exposure (Paunović et al., 2014). Studies have also reported that noise negatively impacts mental health (Hammersen et al., 2016; Jensen et al., 2018), which interacts with a wide range of complex elements, including biological, psychological, social, economic, and environmental factors (Barry and Friedli, 2010; Aletta et al., 2017). These factors include not only objectively measured environmental conditions but also subjective evaluation. When the noise level can no longer be reduced, people can still be annoyed by the noise because their subjective feelings can be affected by other psychoacoustic attributes, such as sharpness and roughness (Zwicker and Fastl, 1999). Noise and noise annoyance have non-standard effects on individuals that might depend on previous experiences or biological susceptibility. When individuals do not have control over the noise, as experienced with noise annoyance, they might suffer from learned helplessness and biological signatures of chronic stress, including overproduction of cortisol (Recio et al., 2016).

Since the formulation of eco-effective design (EED) and evidence-based design, the restorative effects of the environment have attracted wide attention (Ulrich et al., 2008; Shepley et al., 2009). As the primary facility for helping people to recover from illness, hospitals have also begun to focus on developing a healthy spatial environment utilizing natural forces. Through studying soundscapes, sounds in the environment have been regarded as a useful resource, and a favorable and healthy spatial environment can be created through discussing human perception and experience (Kang et al., 2016). As a result of people’s perception of the acoustic environment, soundscapes can be positive (such as happy, calm) or negative (such as worry, pressure). The research on the effect of soundscape restoration is based on the development of attention restoration theory (ART) proposed by Kaplan and Kaplan (1989) and stress restoration theory (SRT) proposed by Ulrich et al. (1991). Weakening negative soundscapes is significantly related to health status, and increasing positive soundscapes is significantly related to environmental pressure recovery (Aletta et al., 2018a, b). A series of previous studies revealed that design and occupant choices can have positive health impacts by controlled reduction of noise levels (Evans, 2003; Von Lindern et al., 2016; Aletta et al., 2018c). It was also found that the natural environment had a positive effect on restoration processes (Hartig and Staats, 2003). However, the restorative effects of soundscapes should be correlated not only with subjective evaluation data but also with physiological parameters, including the emotions caused by sound stimulation (Hume and Ahtamad, 2013; Aletta et al., 2016a). Moreover, the soundscape is related to other spatial environmental factors. When people hear a sound, the perceived auditory space around them may modulate their emotional response to it. Small rooms are considered to be more pleasant, calmer, and safer than large rooms, and sounds originating behind listeners tend to be more arousing and elicit larger physiological changes than sources in front of the listeners (Tajadura-Jiménez et al., 2010). In their work on soundscapes in hospitals, researchers have revealed the relationship between the acoustic environment, typical sound sources, and geometry form (Xie and Kang, 2012b). An acoustic environment evaluation system has also been established (Xie and Kang, 2012a), and it has been found that the acoustic environment plays a leading role in the overall environmental evaluation (Wu et al., 2019). However, the impacts of hospital acoustic soundscapes on the physiological and psychological indices of patients require further study.

Perceptual experiences in one modality often depend on activity from other sensory modalities. The renewed interest in the topic of cross-modal correspondences that have emerged in recent years has motivated research that demonstrated that cross-modal matchings and mappings exist between most sensory dimensions (Deroy and Spence, 2016). Individuals reliably match different tastes/flavors (KnFerle and Spence, 2012), colors (Hamilton-Fletcher et al., 2016), and shapes (Ozturk et al., 2013) to auditory stimuli. For example, individuals consistently match high-pitched sounds to small, bright objects located high up in space (Spence, 2011). In each experimental module, participants were experiencing different hospital indoor environments as the different experimental scenario conditions. Experimental scenarios can be classified as real or artificial. Due to site restrictions, it is difficult to effectively control a large number of irrelevant environmental factors, and it is also difficult to “add” a new environmental factor to the original indoor environment. Therefore, the experimental conditions of real scenarios are limited by their controllability (Stamps, 2007). To gain the most accurate and least variable estimate of acoustic environmental stimuli/properties, the stimulation of other environmental factors should be minimized. With the increasing maturity of virtual reality (VR) technology in recent years, VR environments can provide users with a more realistic and immersive environment (Chamilothori et al., 2019a). Multiple empirical studies show that the physiological, psychological, and behavioral feedback of participants in VR scenarios is similar to those in real scenarios (Heydarian et al., 2015). Yin et al. (2018) found that, in VR scenarios, the user’s heart rate, blood pressure, skin conductivity, cognitive ability, and emotional level were very similar to those in real scenarios. Therefore, environmental psychologists began to use VR scenarios for environmental psychology experiments, rather than real scenarios.

### Study Framework

This study aims to determine the following: (1) whether the acoustic environment can promote recovery in terms of physiological indicators—we hypothesize that physiological recovery will increase with music and decrease with artificial sounds and mechanical sounds; (2) whether sounds can decrease or increase the psychological function of patients in hospital wards—we hypothesize that music will be helpful for the psychological restoration of patients, as artificial and mechanical sounds will lead to the opposite trend; and (3) whether demographic factors and other environmental factors will cause different degrees of impact—we hypothesize that differences in demographic and environmental factors will lead to differences in the degree of the effect of soundscape recovery, as some previous studies indicated that there are differences between population and other environmental factors in the subjective evaluation of the acoustic environment. A digital three-dimensional (3D) model of a room was constructed, and experimental patients wore VR glasses to visualize the same ward scene and eliminate other visual and landscape distractions. Several different approaches were explored to meet the goals. First, the effect of sound stimuli on the physiological indices of the patients was examined. Second, the effect of sound stimuli on an individual’s mental health was examined. Third, differences in the effects of sound on different populations and multiple environmental interactions were observed.

## Methodology

In this study, a combination of physiological measurements and psychological evaluation was utilized. Four typical sound types were presented to experimental patients, and their physiological indicators were monitored by attached detectors. The patients wore VR glasses to observe the same virtual ward space and eliminate interference from other environments. The participants were asked to complete a subjective questionnaire. The obtained data were analyzed to evaluate the restorative effect of sounds in hospitals on individuals utilizing statistical methods.

### Participants

The participants were all inpatients of the internal medicine department of the First Hospital of Harbin and the Second and Fourth Affiliated Hospitals of Harbin Medical University. Inpatients from internal medicine tend to have more time to participate in experiments than outpatients, and internal medicine is mainly related to chronic diseases; this provides an ideal experimental object that can exclude the psychological and physiological effects of diseases.

The participants were 70 patients with an average age of 48.2 (*SD* = 3.42; min = 18; max = 72), including 36 men and 34 women. The proportions of participants younger than 45, 45–60, and over 60 years old were similar to remove the effects of differences between participant groups on the experimental results (Zhang et al., 2016). The number of participants selected in this study was based on relevant experiments conducted in similar fields (Alvarsson et al., 2010; Annerstedt et al., 2013).

All participants were required to have clear cognitive consciousness and sufficient visual, auditory, and behavioral abilities to ensure that they could complete the physiological index measurement and questionnaire survey, and wore comfortable clothing. Additionally, patients with hyperthyroidism and supraventricular tachycardia were excluded from this experiment, as autonomic nervous dysfunction would decrease the accuracy of the measurement and evaluation of physiological stress indicators. The diet and sleep status of the participants needed to be stable. Six hours before the test, the patients did not drink, smoke, or have coffee or other drinks that would stimulate the sympathetic nervous system (Li and Kang, 2019).

The study was approved by the professors’ associates in the School of Architecture at Harbin Institute of Technology. Written informed consent was obtained from all participants before the test began. Participants were informed about the goals and contents of the study, privacy, and data protection and that their participation in the study was voluntary. Biological samples were not collected.

### Visual Scene

**FIGURE 1:**
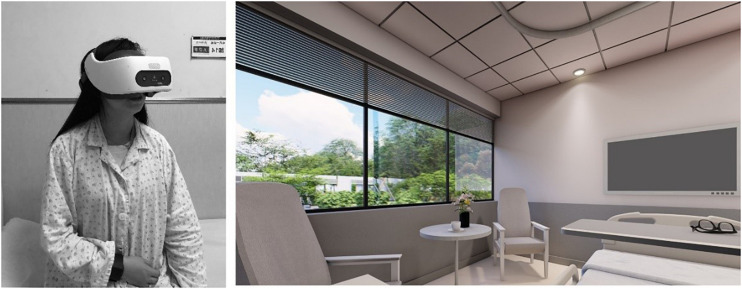
Virtual reality (VR) device HTC Vive Focus Plus and presenting virtual scene. **(A)** Virtual scene of the ward. **(B)** Participant wearing VR.

Many studies have been conducted on the influence of audio-visual factors on noise perception (Collignon et al., 2008; Yost and Zhong, 2015; Aletta et al., 2016b; Liu and Kang, 2018), and it has been demonstrated that vision and hearing can influence one other. Therefore, to prevent other factors influencing the patients’ psychological and physiological indices, participants wore VR glasses and observed the same ward scene. A standard single ward was selected as the experimental scene, as shown in Figure 1A. The simulated ward was 6.6 × 3.6 × 2.8 m, and the bed size was 2.1 × 0.9 m. A U-shaped rail curtain and 0.45 × 0.45 m bedside cabinet were set around the bed. Participants experienced the scene from the perspective of sitting on the bed in the ward and wore VR headsets, as shown in Figure 1B. The transformation of the virtual environment was based on the experimental condition transformation path of basic model construction–experimental parameter adjustment–virtual scene generation. First, a digital 3D model of the indoor space was established by 3DMAX. Then, according to the specific experimental goal, some of the design parameters of the model scenario were adjusted. Finally, the adjusted model was imported into the HTC Vive VR device.

### Selection of Sound Stimuli

Common hospital sound sources can be summarized into four typical categories: mechanical, artificial, background, and music (Rashid and Zimring, 2008). Mechanical sounds are produced by hospital equipment, such as wheelbarrows, ventilators, and electrocardiograph monitors. Artificial sounds include patient conversations, children’s crying, phones being answered, and other behaviors. Background sounds have no clear dominant source and include mechanical sounds produced by new air systems, elevator operations, and other equipment, as well as artificial sounds produced by the conversations and movements of doctors and patients. Mechanical, artificial, and natural sounds were recorded in the participants’ hospital. According to Baker’s classification standard of hospital background noise, the background should be stable, with its amplitude changing less than once per minute (<5 dB) (Baker, 1992). “Day and Night” was selected for the music stimulus, which is a particular piece of music that has been widely used in sound masking systems in hospitals (Ferguson et al., 1997; Mlinek and Pierce, 1997), and studies have demonstrated that it is popular among patients and played a positive role in their well-being, making them feel less tense, more relaxed, and safe (Thorgaard et al., 2004). “Day and Night” was favored by most inpatients, with 82% of the patients being very pleased/pleased with the song and 91% of participants defining the sound environment as very pleasant/pleasant (Bitten et al., 2017). Light music without lyrics was selected for this experiment, which avoids experimental deviation caused by the influence of lyrics on patients and their mental health (Baker and Bor, 2008).

The samples used in the experiments were recorded by SQuadriga II with BHS I, and the type of all four sound source samples can be clearly identified. A 5 min sample without dominant sound sources and only ambient noise was recorded for the control group. Five minutes of representative footage from each recording was used as the stimulation material for the experiment, as prolonged use of a VR headset would cause the subjects to become uncomfortable and interfere with the experimental results (Li and Kang, 2019). The 5 min equivalent sound pressure level (SPL) was adjusted to 50 dB(A) (Liu and Kang, 2018) for each audio frequency by AuditionCS6 to remove differences in volume during the stimulation of the four sounds. To ensure that the participants listened to the four auditory stimulus sounds under similar playback SPL conditions, the LAeq of the audio stimuli had been normalized by an artificial head to 50 dB(A) before the experiments to exclude the effect on arousal due to loudness. The background noise was below 45 dB(A) during the acoustic stimulation experiment, and nobody spoke in the room. Subjective loudness evaluation was carried out simultaneously; the results show that the loudness levels of different groups are significantly different due to the difference of their dominant source frequencies, but the loudness levels of different participants in the same group can be ignored.

### Measurements

By using VR glasses to observe the 3D virtual hospital ward environment created by virtual simulation and headphones to listen to the four types of sound, participants can experience a more realistic hospital environment. The physiological recovery indices include heart rate and skin conductance, which were measured using the Empatica E4 physiological information monitoring equipment. Information regarding psychological recovery was obtained using a questionnaire. The scale is composed of two parts. The first is psychological feedback, including the anxiety state and perceived environmental restorativeness of the subjects. The anxiety states of participants were measured by using STAI-Y6 with eight questions to indicate their anxiety level (Zijlstra et al., 2017). Perceived restorativeness score (PRS) was adopted to evaluate the subjects’ perceived restorativeness (Hartig et al., 1997). The second part refers to environmental appraisal. Perceived environmental quality index (PEQI) was used to describe the participants’ perceived environmental quality (Fisher, 1974). The scale consists of a set of bipolar adjectives rated on a seven-point Likert scale, ranging from 1 (extreme negative perception of the environment) to 7 (extreme positive perception of the environment).

### Procedure

**FIGURE 2:**
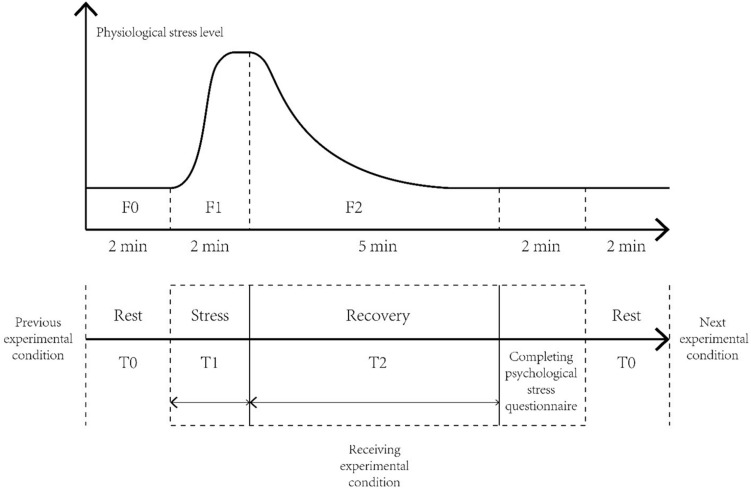
Experimental process.

The participants were asked to sit comfortably on a bed. The investigator explained the entire experimental procedure and asked the participants about their physical and mental states. After the subjects understood and agreed to all the terms, the investigator connected the HTC Vive Focus Plus VR device and Empatica E4. The experiment was started after the completion of the connection process and calibration of the physiological signal. The experimental process is shown in Figure 2. First, stress was induced in the subjects using the PASAT (packed audit serial addition task) program. The subjects then received a sound clip (one of the four types of sound sources), and the indoor scene was displayed by the VR equipment. After receiving one experimental condition, the subjects temporarily removed the VR equipment and completed the psychological recovery questionnaire, which took approximately 2 min. The subjects then rested for 2 min, accepted the next sound clip, and followed the process until the four experimental conditions were completed. The sequence of the four experimental conditions in the experiment followed a Latin square design (Morsbach et al., 1986). The Empatica E4 equipment continuously recorded the physiological recovery index data of the participants.

### Data Analysis

Regarding physiological data transformation, the heart rate and skin conductance, which are the basic physiological indices of the human body, are easily affected by the physical differences between patients. For example, some individuals may have a relatively high basal heart rate or exhibit more intense physiological responses under the PASAT. Therefore, the physiological stress recovery level of participants cannot be compared by the mean values of physiological indices. In this study, we used the standardized physiological stress recovery rate (*R*) to estimate the influence of the acoustic environment on the individuals’ physiological indices to reduce the potential experimental error caused by physical difference (Payne, 2013; Medvedev et al., 2015; Watts et al., 2016). As shown in formula (1), the *R*-value can be obtained by dividing the stress recovery level (the difference between the mean values of *F*1 and *F*2) by the stress arousal level (the difference between the mean values of *F*1 and *F*0). RHR and RSCL represent the stress recovery of the heart rate and skin conductance level, respectively. A higher *R*-value indicates that, under the experimental conditions, the subjects recover from physiological stress faster.

(1) $$R=\frac{\bar{F_{1}}-\bar{F_{2}}}{\bar{F_{1}}-\bar{F_{0}}}$$

For statistical analysis, IBM SPSS 25.0 was used to construct a database containing the final results (Meng et al., 2018; Ba and Kang, 2019). The data were analyzed by the following methods: (1) The differences between the physiological and psychological indicators measured at different times and for different sound source types were determined by repeated analysis of variance measurements, and the level of significance was set at *p* < 0.05. (2) Least significant difference (LSD) *post hoc* tests were conducted for pairwise comparisons. The effect sizes (partial η^2^) were regarded as minimum, intermediate, and high at thresholds of 0.01, 0.06, and 0.14, respectively.

## Results

### Effects of the Acoustic Environment on Physiological Stress Recovery

**FIGURE 3:**
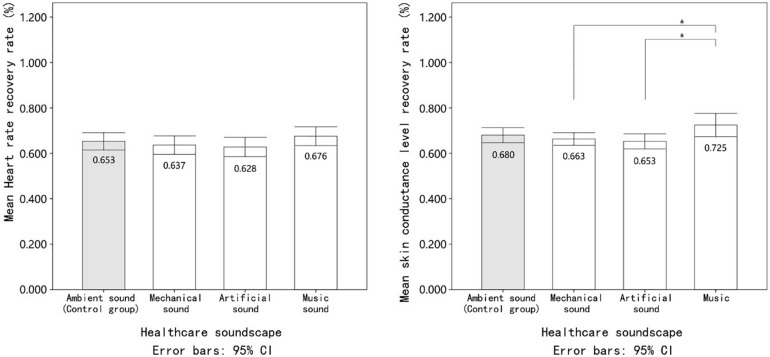
The mean value of patients’ physiological stress recovery indicators under exposure to different healthcare soundscapes. Error bars depict 95% confidence interval. ^∗^Significant at the 0.05 level. ^∗∗^Significant at the 0.01 level.

The results showed that the patient’s mean heart rate recovery rates (*R*_HR_) under the ambient noise, mechanical sound, artificial sound, and music conditions were 0.66 (*SD* = 0.11), 0.64 (*SD* = 0.11), 0.63 (*SD* = 0.12), and 0.68 (*SD* = 0.12), respectively. As shown in Figure 3, the patients’ heart rates tended to recover faster under the music soundscape than the others. However, the repeated measures ANOVA results indicated that the main effect of the soundscape on heart rate recovery was not significant (*F* = 1.35, *p* = 0.26, partial η^2^ = 0.04).

As assumed, the highest skin conductance level recovery rate (*R*_SCL_; *M* = 0.73, *SD* = 0.14) was observed when the patients were exposed to the music soundscape condition. In contrast, the *R*_SCL_ of patients decreased by 2.5 and 4.6% under the mechanical and artificial noise conditions, respectively, when compared to the control group (ambient noise). The main effect of the soundscape on *R*_SCL_ was statistically significant (*F* = 3.37, *p* = 0.02), indicating that there was a significant difference in *R*_SCL_ during exposure to various experimental conditions. Additionally, according to the effect size exhibited by partial η^2^(0.10), the soundscape could exert a substantial impact on *R*_SCL_. In the multiple comparisons, a Bonferroni correction is applied. Given there are six sub-groups, 0.0083 is used as a corrected significance threshold. As shown in Table 1, the results of the LSD *post hoc* test confirmed that the recovery of the skin conductance level was faster under the music soundscape than under the mechanical (*MD* = 0.07) and artificial (*MD* = 0.08) noise conditions. Finally, although exposure to mechanical and artificial noise may lead to slower skin conductance level recovery than ambient noise, the difference was not significant. Combining the results of *R*_HR_ and *R*_SCL_ partly confirms our first hypothesis; i.e., the healthcare soundscape could impact the physiological stress recovery response of patients but only in the case of skin conductance level.

**TABLE 1:** Pairwise comparison of physiological stress recovery levels in patients.

	Heart rate recovery rate				Skin conductance level recovery rate			
		
	MD	95% CI for difference		Sig	MD	95% CI for difference		Sig
					
		Lower bound	Upper bound			Lower bound	Upper bound	
Ambient sounds–mechanical sounds	0.02	–0.03	0.06	0.48	0.02	–0.02	0.06	0.40
Ambient sounds–artificial sounds	0.03	–0.02	0.07	0.27	0.03	–0.02	0.07	0.24
Ambient sounds–music sound	–0.02	–0.08	0.03	0.42	–0.05	–0.09	0.01	0.07
Mechanical sounds–artificial sounds	0.01	–0.04	0.05	0.70	0.01	–0.03	0.05	0.59
Mechanical sounds–music sound	–0.04	–0.10	0.02	0.19	–0.07	–0.12	–0.01	0.03(*)
Artificial sounds–music sound	–0.05	–0.10	0.01	0.09	–0.08	–0.14	–0.01	0.03(*)

* The mean difference is significant at the 0.05 level.

### Effects of the Acoustic Environment on Psychological Stress Recovery

The repeated measures ANOVA results suggested that the main effect of healthcare soundscapes on the anxiety state of patients was statistically significant (*F* = 10.95, *p* = 0.00, partial η^2^ = 0.26). Music soundscapes could effectively reduce the patients’ anxiety state. After experiencing the music soundscape, the anxiety states of the participants were 16.7, 14.4, and 24.5% lower than those under the ambient, mechanical, and artificial noise conditions, respectively. The LSD *post hoc* test indicated that these differences all reached statistical significance (*p* < 0.008). Additionally, artificial noise could cause patients to feel more anxious than the other three soundscapes. As shown in Table 2, the LSD *post hoc* test revealed that the difference in the anxiety score between mechanical noise and artificial noise was significant (*p* = 0.007), but that between the mechanical and control groups was not (*p* = 0.0027).

**TABLE 2:** Pairwise comparison of psychological stress recovery levels in patients.

	Self-reported anxiety state				Perceived restorativeness score			
		
	MD	95% CI for difference		Sig	MD	95% CI for difference		Sig
					
		Lower bound	Upper bound			Lower bound	Upper bound	
Ambient sounds–mechanical sounds	0.25	–0.55	1.05	0.530	2.09	0.28	3.91	0.04(*)
Ambient sounds–artificial sounds	–0.97	–1.82	–0.12	0.027(*)	0.78	–1.04	2.60	0.35
Ambient sounds–music sound	1.56	0.52	2.61	0.01(**)	–2.69	–4.51	–0.87	0.00(**)
Mechanical sounds–artificial sounds	–1.22	–2.08	–0.36	0.01(**)	–1.32	–3.13	0.51	0.20
Mechanical sounds–music sound	1.31	0.46	2.17	0.00(**)	–4.78	–6.60	–2.96	0.00(**)
Artificial sounds–music sound	2.53	1.52	3.54	0.00(**)	–3.46	–5.29	–1.65	0.00(**)

* The mean difference is significant at the 0.05 level. ** The mean difference is significant at the 0.01 level. Values in bold represent the family-wise error rate corrected significant.

**FIGURE 4:**
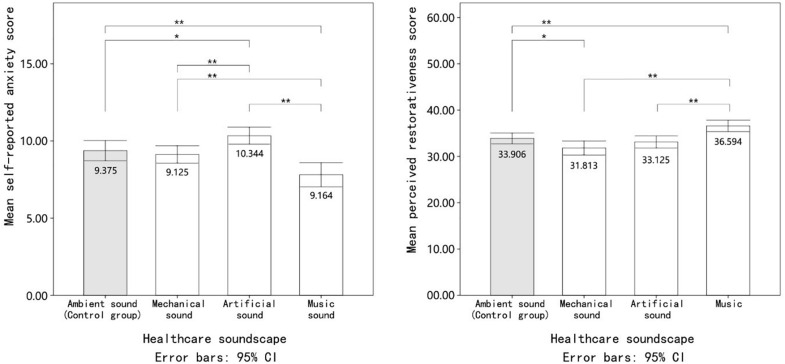
The mean value of patients’ psychological stress recovery indicators under exposure to different healthcare soundscapes. Error bars depict 95% confidence interval. *Significant at the 0.05 level. **Significant at the 0.01 level.

The environmental restorativeness scores given by patients significantly differed (*F* = 9.39, Sig = 0.00) under the ambient noise, mechanical noise, artificial, and music soundscape conditions. The effect size (partial η^2^ = 0.23) suggested the substantial effect of the healthcare acoustic environment on the perceived environmental restorativeness scores. As shown in Figure 4, consistent with the anxiety result, when the music soundscape was broadcast, patients tended to perceive the surrounding environment as “restorative.” The percentage of improvement in the restorativeness scores, *i*, was used to estimate the benefit that certain soundscapes could bring to the participants, which can be calculated as follows: *i*music = (PRSmusic − PRSnoise)/PRSnoise, where *i*music is the percentage of improvement in the restorativeness scores from the music to the noise soundscape conditions, PRSmusic is the mean PRS under music soundscape conditions, and PRSnoise is the mean PRS under noise soundscape conditions. The restorativeness scores given to the music condition were 7.9, 15.0, and 10.5% higher than those given to the ambient, mechanical, and artificial noise conditions, respectively. The LSD *post hoc* test indicated that the restorativeness differences between music and the other three conditions were all significant (*p* < 0.008). In contrast, participants regarded mechanical noise as the least restorative soundscape (*M* = 31.81, *SD* = 4.20), but the difference between mechanical noise and the control group was insignificant.

**FIGURE 5:**
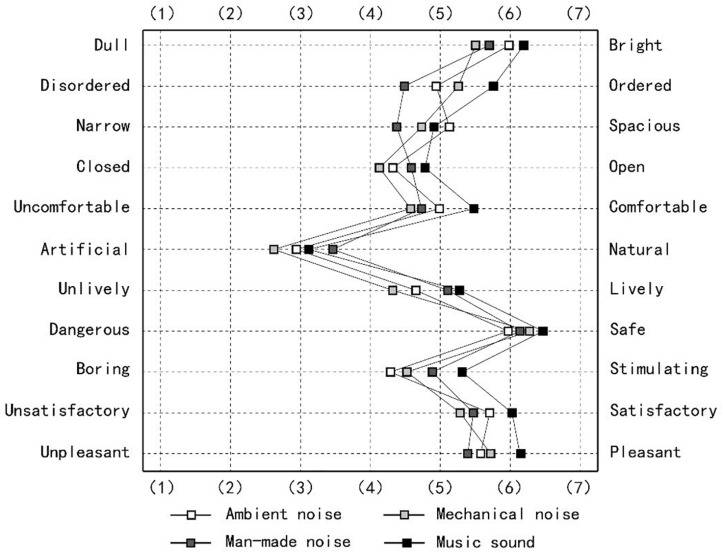
Patients’ bipolar adjective environmental appraisal scores in different healthcare acoustic environments.

The repeated measures ANOVA results showed that the main effects of healthcare soundscapes on the three environmental appraisal indices, i.e., sense of order, comfort, and stimulation, were significant. Under the music experimental condition, patients perceived the virtual environment as more orderly, comfortable, and stimulating (Figure 5). In contrast, participants experiencing the mechanical noise condition were more likely to use negative adjectives to describe the sound than under the control conditions. Specifically, when patients were exposed to mechanical noise, they evaluated the surrounding environment as narrower, closed, uncomfortable, artificial, and unlively. However, the difference was not significant in any of the environmental appraisal indices. Additionally, healthcare soundscapes may influence some visual environmental appraisal parameters. For example, the results indicated that patients considered the space to be more dull and narrow under artificial and mechanical noise, respectively.

### Interaction Effects of the Acoustic Environment and Demographic Factors on Stress Recovery

**FIGURE 6:**
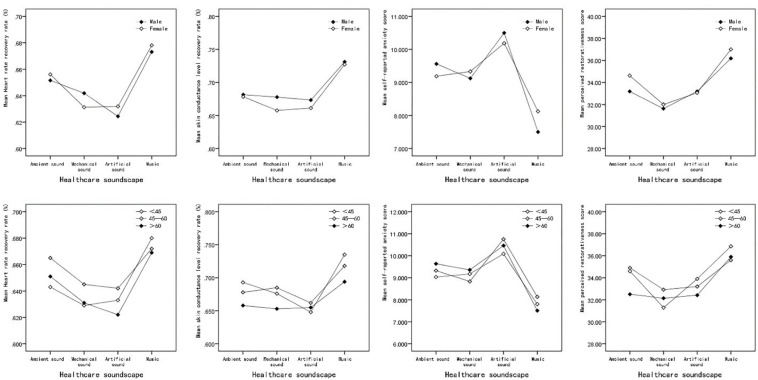
**(A)** The interaction effects of gender and acoustic environment on patients’ stress recovery. **(B)** The interaction effects of age and acoustic environment on patients’ stress recovery.

As shown in Figure 6, mechanical noise appeared to exert a more negative impact on physiological stress recovery in female patients. Under the mechanical noise condition, the mean *R*_HR_ and *R*_SCL_ values of female patients were 1.68 and 3.09% lower than those of male patients. However, the ANOVA results showed that the main effects of gender on *R*_HR_, *R*_SCL_, anxiety state, and perceived restorativeness state were not significant (*p* < 0.05). In addition, there was no significant interaction effect between gender and healthcare soundscape on physiological and psychological stress recovery, indicating that the street recovery outcomes of male and female patients in response to various healthcare soundscapes were similar.

As suggested by Figure 6, patients of different age groups tended to respond to different healthcare soundscapes similarly, although patients aged between 45 and 60 appeared to physiologically recover slightly faster than those in the other age groups. A two-way repeated measures ANOVA was conducted to examine the influence of age on the participants’ physiological and psychological stress recovery parameters, and the results indicated that neither the main effect of the patients’ age nor the interaction effect between age and soundscape condition was significant (*p* < 0.05), as shown in Table 3. The interaction effect between age and soundscape on the perceived restorativeness was almost significant (*p* = 0.05). Senior patients were less sensitive to the three types of healthcare noise, and young people (less than 45 years old) perceived the environment as less restorative under the mechanical noise condition.

**TABLE 3:** MANOVA results for main and interaction effects on physiological and psychological recovery.

	Physiological stress recovery				Psychological stress recovery			
		
	*R*_HR_		*R*_SCL_		STAI		PRS	
				
	*F*	Sig	*F*	Sig	*F*	Sig	*F*	Sig
Gender	0.01	0.94	0.30	0.59	0.01	0.91	0.91	0.34
Gender*soundtype	0.04	0.99	0.05	0.99	0.57	0.64	0.26	0.86
Age	0.23	0.79	2.00	0.19	0.28	0.73	0.82	0.45
Age*soundtype	0.36	0.91	1.07	0.36	1.78	0.10	2.14	0.05

The results showed that there was no significant interaction effect between soundscape and demographic characteristics on the participants’ restoration; therefore, hypothesis (3) is rejected. Although we failed to identify any significant interaction effects, certain groups of participants exhibited some particular environmental feedback tendencies. For example, the restorative outcome of elderly people appeared to be less sensitive to acoustical conditions. Participants aged between 45 and 60 years tended to withstand negative sounds better than those in the other age groups. In future studies, we could consider the patients’ socioeconomic characteristics in analysis to explore the potential interaction effects.

## Discussion

The results obtained from the skin conductance level partly support the theory that the healthcare soundscape could affect physiological stress recovery. The music soundscape has a restorative effect on healthcare. The results showed that the recovery rate of participants’ SCL under the music condition was faster than that under the ambient, mechanical, and artificial sound conditions. However, none of these differences reached significance, indicating that the restorative effects of music are limited in the aspect of physiological stress.

Some previous studies observed a stronger restorative effect of music than this study (Medvedev et al., 2015). The experimental setting we adopted may cause different results, as VR conditions could draw the patients’ attention to visual stimuli and weaken the restorative effect of soundscapes. Another possible explanation may be that the music used in this study was not self-selected. Researchers have confirmed that a participant’s sense of control could improve restorative effects (Heitz et al., 1992). Additionally, the different musical tastes of patients could affect the restorative impact (Watts et al., 2016).

No significant impact of soundscape on the patients’ *R*_HR_ was observed here, which is consistent with the results of some previous studies (Aletta et al., 2018a; Yu et al., 2018). This may be attributed to the special characteristics of heart rate, which is highly sensitive to the mode of information processing (Ulrich et al., 1991; Meehan et al., 2005). A person’s heart rate accelerates dramatically if experimental conditions involve information processing, such as mental counting (Lacey and Lacey, 1974). This study involved no information storage, retrieval, or manipulation under any of the four soundscapes, which may have resulted in an insignificant effect on heart rate. Additionally, although the skin conductance level and heart rate are both indicators of sympathetic nervous system activity, their sensitivities when testing various built environmental stimuli may differ (Cacioppo et al., 2007). Studies have found that skin conductance is sensitive to changes in the luminous environment (Izso et al., 2009) and that heart rate is more responsive to the facade pattern (Chamilothori et al., 2019a).

The second hypothesis was confirmed by the significant effects of soundscape on the participants’ anxiety level and perceived restorativeness state. Artificial sound induced higher anxiety than the mechanical experimental conditions in the study. A potential explanation for this result is that artificial sound contains more transient noise (Allaouchiche et al., 2002), which may negatively impact psychological recovery. However, the anxiety level of the participants experiencing the mechanical sound was not significantly higher than that of the participants in the control group. Although this is the first study in this area, the influence of the participants’ acoustic expectations may explain this result. The potential function of space (such as socializing and working) could influence a user’s environmental expectations and evaluations (Chamilothori et al., 2019b). In this study, healthcare was chosen as the research context, and participants may anticipate certain kinds of mechanical noise before being subjected to the experimental conditions. Thus, its negative impact on recovery could be relieved.

The perceived restorativeness state was also significantly influenced by healthcare soundscapes. Mechanical noise was perceived as the least restorative condition in the study, which was inconsistent with the anxiety state data. This might be due to the different assessment weights between the two psychological recovery indicators. The anxiety state assesses the participants’ mental state. For example, the scale we used included items such as “I feel upset” (Marteau and Bekker, 1992). However, the perceived restorativeness state reflects the external environment appraisal. In this study, both parameters may partly reflect the impact of the soundscape on the patients’ psychological states, but more work should be conducted to determine the mechanisms and pathways of the effects of healthcare soundscapes on psychological stress recovery.

This research assessed the effects of the acoustic environment on patients’ environmental appraisal in a healthcare facility, and the data show that healthcare soundscapes may have affected the participants’ environmental appraisal. The music condition was perceived as being more positive than the three other experimental conditions for nine of the 11 evaluation dimensions and could significantly improve the patients’ environmental perception in terms of the order, comfort, and stimulation. However, no significant difference was observed between the evaluation of mechanical, artificial, and ambient noise. Overall, the soundscape has less of an impact on patients’ environmental appraisal than visual information, such as color, lighting, and spatial layout (Leather et al., 2003). The smaller effect may indicate that visual stimulation is a dominating factor affecting environmental appraisal in healthcare settings.

This study found that the soundscape could alter the patients’ visual impression of the environment, such as their sense of light, order, and scale. This could be because people holistically perceive the environment, and audio and visual stimuli could drive multisensory environmental perception (Viollon et al., 2002; Pheasant et al., 2010). Attractive or meaningful visual contexts tend to increase people’s tolerance to noise, causing less irritation in similar noisy acoustic environments (Bangjun et al., 2003; Iachini et al., 2012). However, studies have also observed a high correlation between audio information and an individual’s visual experience and preference. In this study, the participants tended to regard the surrounding environment as orderly, comfortable, and stimulating under the music condition, which may be because the sound stimuli altered the participants’ visual cognitive processing (Ren and Kang, 2015).

Generally, the objective data were relatively consistent with the subjective ratings (anxiety, perceived restorativeness state, and environmental appraisal), which could verify the validity of the method used in the study. However, when faced with audio stimuli, the psychological stress recovery indicator tended to be more sensitive than physiological parameters. The effect size also indicates that the soundscape could exert a greater effect on the outcome of psychological factors, supporting the results of previous studies. This may be because physiological stress recovery parameters, such as heart rate, skin conduction levels, or blood pressure, are indices of sympathetic arousal and cannot reflect the valence of emotion (Ward and Marsden, 2003). Therefore, physiological data cannot detect mild arousal responses coupled with positive emotional reactions. Therefore, interpreting a person’s stress level using physiological data alone is insufficient, especially considering the stress reaction, and recovery is a complex process involving cognition and reflection (Bartlett, 1998).

## Conclusion

Inpatients are more prone to anxiety and stress than healthy individuals; therefore, hospital wards must provide suitable acoustic environments to help them to relax and recover. The restorative effects of soundscapes have been investigated, but few studies have been conducted on patients and hospital environments. This study mainly explores and analyzes the influence of the acoustic environment on physiological/psychological stress indicators of patients in hospital wards.

The impact of soundscape on patients’ physiological stress parameters was relatively modest. In this study, sounds did not significantly impact the patients’ heart rate recovery rates (*R*_HR_). However, the results demonstrate that the soundscape could significantly influence the patients’ skin conductance level recovery rate (*R*_SCL_). The recovery rate was faster under music than the mechanical or artificial noise conditions, though the difference fails to reach significance.

The acoustic environment could exert profound effects on the patients’ psychological stress indicators, with both the patients’ self-reported anxiety state and PRS significantly affected by the healthcare soundscapes. Patients continuously reported less anxiety and higher perceived restorativeness for the music soundscape than the ambient, mechanical, and artificial noise soundscapes. The reported anxiety levels were highest under the artificial sounds, and mechanical sounds were regarded as the least restorative. For the environmental appraisal of psychological parameters, the music condition was described as significantly more ordered, comfortable, and stimulating than the three other experimental conditions. There was no statistically significant difference between the environmental appraisal of mechanical, artificial, and ambient sounds. However, it was found that the acoustic environment could alter the patients’ visual impression of the environment.

The interaction effects of gender, age, and acoustic environment were not significant. However, there were some environmental feedback tendencies for certain groups of participants, and future studies may consider the patients’ social–economic characteristics. Hospital spaces are rather diverse; thus, it would be interesting to consider other spaces, such as outpatient halls, waiting rooms, double beds, and dormitory bed wards, in future studies. While this study indicated that the acoustic environment of hospital wards influences the physiological and psychological indices of patients, and also demonstrated that VR is an effective method of analyzing the relative influences of different dominant sound sources, in future work, the absolute influence of the acoustic environment on the psychological and physiological indicators of patients could be examined in realistic environments.

## Data Availability Statement

The original contributions presented in the study are included in the article/supplementary material. Further inquiries can be directed to the corresponding author/s.

## Ethics Statement

Ethical review and approval was not required for the study on human participants in accordance with the local legislation and institutional requirements. Written informed consent to participate in this study was provided by the participants. Written informed consent was obtained from the individual for the publication of any potentially identifiable images or data included in this article.

## Author Contributions

TZ, YW, QM, and JK contributed to conceptualization, methodology, and visualization. TZ contributed to investigation, formal analysis, and data curation. TZ and YW contributed to writing original draft preparation. TZ contributed to review and editing. YW, QM, and JK contributed to supervision. YW and QM contributed to administration and funding acquisition. All authors contributed to the article and approved the submitted version.

## Conflict of Interest

The authors declare that the research was conducted in the absence of any commercial or financial relationships that could be construed as a potential conflict of interest.

## References

[B1] Aletta F. Axelsson Ö. Kang J. (2017). Dimensions underlying the perceived similarity of acoustic environments. *Front. Psychol.* 8:1162. 10.3389/fpsyg.2017.01162 28747894 PMC5506192

[B2] Aletta F. Kang J. Axelsson Ö. (2016a). Soundscape descriptors and a conceptual framework for developing predictive soundscape models. *Landsc. Urban Plan.* 149 65–74. 10.1016/j.landurbplan.2016.02.001

[B3] Aletta F. Masullo M. Maffei L. Kang J. (2016b). The effect of vision on the perception of the noise produced by a chiller in a common living environment. *Noise Control Eng. J.* 64 363–378. 10.3397/1/3763786

[B4] Aletta F. Oberman T. Kang J. (2018a). Associations between positive health-related effects and soundscapes perceptual constructs: a systematic review. *Int. J. Environ. Res. Public Health* 15:2392. 10.3390/ijerph15112392 30380601 PMC6266166

[B5] Aletta F. Oberman T. Kang J. (2018b). Positive health-related effects of perceiving urban soundscapes: a systematic review. *Lancet* 392:S3. 10.3390/ijerph15112392 30380601 PMC6266166

[B6] Aletta F. Van Renterghem T. Botteldooren D. (2018c). Influence of personal factors on sound perception and overall experience in urban green areas. A case study of a cycling path highly exposed to road traffic noise. *Int. J. Environ. Res. Public Health* 15:1118. 10.3390/ijerph15061118 29848994 PMC6025617

[B7] Allaouchiche B. Duflo F. Debon R. Bergeret A. Chassard D. (2002). Noise in the postanaesthesia care unit. *Br. J. Anaesth.* 88 369–373. 10.1093/bja/88.3.369 11990268

[B8] Alvarsson J. J. Wiens S. Nilsson M. E. (2010). Stress recovery during exposure to nature sound and environmental noise. *Int. J. Environ. Res. Public Health* 7 1036–1046. 10.3390/ijerph7031036 20617017 PMC2872309

[B9] Annerstedt M. Jönsson P. Wallergård M. Johansson G. Karlson B. Grahn P. (2013). Inducing physiological stress recovery with sounds of nature in a virtual reality forest — Results from a pilot study. *Physiol. Behav.* 118 240–250. 10.1016/j.physbeh.2013.05.023 23688947

[B10] Ba M. Kang J. (2019). Effect of a fragrant tree on the perception of traffic noise. *Build. Environ.* 156 147–155. 10.1016/j.buildenv.2019.04.022

[B11] Baker C. F. (1992). Discomfort to environmental noise: heart rate responses of SICU patients. *Crit. Care Nurs. Q.* 15 75–90. 10.1097/00002727-199208000-00006 1628246

[B12] Baker F. Bor W. (2008). Can music preference indicate mental health status in young people? *Australas. Psychiatry* 16 284–288. 10.1080/10398560701879589 18608148

[B13] Bangjun Z. Lili S. Guoqing D. (2003). The influence of the visibility of the source on the subjective annoyance due to its noise. *Appl. Acoust.* 64 1205–1215. 10.1016/s0003-682x(03)00074-4

[B14] Barry M. Friedli L. (2010). “The influence of social, demographic and physical factors on positive mental health in children, adults and older people,” in *Mental Capital and Wellbeing*, eds Cooper C. L. Field J. Goswami U. Jenkins R. Sahakian B. J. (Hoboken, NJ: Wiley-Blackwell), 475–484.

[B15] Bartlett D. (1998). *Stress: Perspectives and Processes.* Buckingham: McGraw-Hill Education.

[B16] Belojevic G. Paunovic K. Jakovljevic B. Stojanov V. Ilic J. Slepcevic V. (2011). Cardiovascular effects of environmental noise: research in Serbia. *Noise Health* 13 217–220. 10.4103/1463-1741.80156 21537105

[B17] Berglund B. Lindvall T. Schwela D. H. Organization W. H. (1999). *Guidelines for Community Noise.* Geneva: World Health Organization.

[B18] Beutel M. E. Jünger C. Klein E. M. Wild P. Lackner K. Blettner M. (2016). Noise annoyance is associated with depression and anxiety in the general population-the contribution of aircraft noise. *PLoS One* 11:e0155357. 10.1371/journal.pone.0155357 27195894 PMC4873188

[B19] Bitten T. Birgitte B. H. Gunhild P. Inger T. (2017). Specially selected music in the cardiac laboratory—an important tool for improvement of the wellbeing of patients. *Eur. J. Cardiovasc. Nurs.* 3 21–26. 10.1016/j.ejcnurse.2003.10.001 15053885

[B20] Buckles E. (1990). Evaluation of patient satisfaction in A&E. *Nurs. Stand.* 4 33–35. 10.7748/ns.4.19.33.s40 2107432

[B21] Cacioppo J. T. Tassinary L. G. Berntson G. (2007). *Handbook of Psychophysiology.* Cambridge: Cambridge University Press.

[B22] Chamilothori K. Chinazzo G. Rodrigues J. Dan-Glauser E. Wienold J. Andersen M. (2019a). Subjective and physiological responses to façade and sunlight pattern geometry in virtual reality. *Build. Environ.* 150 144–155. 10.1016/j.buildenv.2019.01.009

[B23] Chamilothori K. Wienold J. Andersen M. (2019b). Adequacy of immersive virtual reality for the perception of daylit spaces: comparison of real and virtual environments. *Leukos* 15 203–226. 10.1080/15502724.2017.1404918

[B24] Collignon O. Girard S. Gosselin F. Roy S. Saint-Amour D. Lassonde M. (2008). Audio-visual integration of emotion expression. *Brain Res.* 1242 126–135. 10.1016/j.brainres.2008.04.023 18495094

[B25] Deroy O. Spence C. (2016). Crossmodal correspondences: four challenges. *Multisens. Res.* 29 29–48. 10.1163/22134808-00002488 27311290

[B26] Evans G. W. (2003). The built environment and mental health. *J. Urban Health* 80 536–555. 10.1093/jurban/jtg063 14709704 PMC3456225

[B27] Farrehi P. M. Nallamothu B. K. Navvab M. (2016). Reducing hospital noise with sound acoustic panels and diffusion: a controlled study. *BMJ Qual. Saf.* 25 644–646. 10.1136/bmjqs-2015-004205 26208539

[B28] Ferguson E. Singh A. P. Cunningham-Snell N. (1997). Stress and blood donation: effects of music and previous donation experience. *Br. J. Psychol.* 88(Pt 2), 277–294. 10.1111/j.2044-8295.1997.tb02635.x 9183841

[B29] Fisher J. D. (1974). Situation specific variables as determinants of perceived environmental aesthetic quality and perceived crowdedness. *J. Res. Pers.* 8 177–188. 10.1016/0092-6566(74)90019-1

[B30] Hamilton-Fletcher G. Ward J. Wright T. D. (2016). Cross-modal correspondences enhance performance on a colour-to-sound sensory substitution device. *Multisens. Res.* 29 337–363. 10.1163/22134808-00002519 29384607

[B31] Hammersen F. Niemann H. Hoebel J. (2016). Environmental noise annoyance and mental health in adults: findings from the cross-sectional German Health Update (GEDA) Study 2012. *Int. J. Environ. Res. Public Health* 13:954. 10.3390/ijerph13100954 27681736 PMC5086693

[B32] Hartig T. Korpela K. Evans G. W. Gärling T. (1997). A measure of restorative quality in environments. *Scand. Hous. Plan. Res.* 14 175–194. 10.1080/02815739708730435

[B33] Hartig T. Staats H. (2003). Guest Editors’ introduction: restorative environments. *J. Environ. Psychol.* 23 103–107. 10.1016/s0272-4944(02)00108-1

[B34] Heitz L. Symreng T. Scamman F. (1992). Effect of music therapy in the postanesthesia care unit: a nursing intervention. *J. Post Anesth. Nurs.* 7 22–31. 1735869

[B35] Heydarian A. Carneiro J. P. Gerber D. Becerik-Gerber B. Hayes T. Wood W. (2015). Immersive virtual environments versus physical built environments: a benchmarking study for building design and user-built environment explorations. *Autom. Constr.* 54 116–126. 10.1016/j.autcon.2015.03.020

[B36] Hume K. Ahtamad M. (2013). Physiological responses to and subjective estimates of soundscape elements. *Appl. Acoust.* 74 275–281. 10.1016/j.apacoust.2011.10.009

[B37] Iachini T. Maffei L. Ruotolo F. Senese V. P. Ruggiero G. Masullo M. (2012). Multisensory assessment of acoustic comfort aboard metros: a virtual reality study. *Appl. Cogn. Psychol.* 26 757–767. 10.1002/acp.2856

[B38] Izso L. Láng E. Laufer L. Suplicz S. Horváth Á. (2009). Psychophysiological, performance and subjective correlates of different lighting conditions. *Light. Res. Technol.* 41 349–360. 10.1177/1477153509336798

[B39] Jensen H. A. Rasmussen B. Ekholm O. (2018). Neighbour and traffic noise annoyance: a nationwide study of associated mental health and perceived stress. *Eur. J. Public Health* 28 1050–1055. 10.1093/eurpub/cky091 29846583

[B40] Kang J. Aletta F. Gjestland T. T. Brown L. A. Botteldooren D. Schulte-Fortkamp B. (2016). Ten questions on the soundscapes of the built environment. *Build. Environ.* 108 284–294. 10.1016/j.buildenv.2016.08.011

[B41] Kaplan R. Kaplan S. (1989). *The Experience of Nature: A Psychological Perspective.* New York, NY: Cambridge University Press.

[B42] KnFerle K. Spence C. (2012). Crossmodal correspondences between sounds and tastes. *Psychon. Bull. Rev.* 19 1–15. 10.3758/s13423-012-0321-z 23055144

[B43] Lacey B. C. Lacey J. I. (1974). “Studies of heart rate and other bodily processes in sensorimotor behavior,” in *Cardiovascular Psychophysiology: Current Issues in Response Mechanisms, Biofeedback and Methodology*, eds Obrist P. A. Black A. H. Brener J. DiCara L. V. (New Brunswick, NJ: Aldine Transaction), 538–564. 10.4324/9781315081762-31

[B44] Leather P. Beale D. Santos A. Watts J. Lee L. (2003). Outcomes of environmental appraisal of different hospital waiting areas. *Environ. Behav.* 35 842–869. 10.1177/0013916503254777

[B45] Li Z. Kang J. (2019). Sensitivity analysis of changes in human physiological indicators observed in soundscapes. *Landsc. Urban Plan.* 190:103593 10.1016/j.landurbplan.2019.103593

[B46] Liu F. Kang J. (2018). Relationship between street scale and subjective assessment of audio-visual environment comfort based on 3D virtual reality and dual-channel acoustic tests. *Build. Environ.* 129 35–45. 10.1016/j.buildenv.2017.11.040

[B47] MacKenzie D. Galbrun L. (2007). Noise levels and noise sources in acute care hospital wards. *Build. Serv. Eng. Res. Technol.* 28 117–131. 10.1177/0143624406074468

[B48] Marteau T. M. Bekker H. (1992). The development of a six-item short-form of the state scale of the Spielberger State—Trait Anxiety Inventory (STAI). *Br. J. Clin. Psychol.* 31 301–306. 10.1111/j.2044-8260.1992.tb00997.x 1393159

[B49] Medvedev O. Shepherd D. Hautus M. J. (2015). The restorative potential of soundscapes: a physiological investigation. *Appl. Acoust.* 96 20–26. 10.1016/j.apacoust.2015.03.004

[B50] Meehan M. Razzaque S. Insko B. Whitton M. Brooks F. P. (2005). Review of four studies on the use of physiological reaction as a measure of presence in stressful virtual environments. *Appl. Psychophysiol. Biofeedback* 30 239–258. 10.1007/s10484-005-6381-3 16167189

[B51] Meng Q. Zhao T. Kang J. (2018). Influence of music on the behaviors of crowd in urban open public spaces. *Front. Psychol.* 9:596. 10.3389/fpsyg.2018.00596 29755390 PMC5934855

[B52] Mlinek E. J. Pierce J. (1997). Confidentiality and privacy breaches in a university hospital emergency department. *Acad. Emerg. Med.* 4 1142–1146. 10.1111/j.1553-2712.1997.tb03697.x 9408430

[B53] Morsbach G. McCulloch M. Clark A. (1986). Infant crying as a potential stressor concerning mothers’ concentration ability. *Psychologia* 29 18–20.

[B54] Okokon E. O. Turunen A. W. Ung-Lanki S. Vartiainen A.-K. Tiittanen P. Lanki T. (2015). Road-traffic noise: annoyance, risk perception, and noise sensitivity in the Finnish adult population. *Int. J. Environ. Res. Public Health* 12 5712–5734. 10.3390/ijerph120605712 26016432 PMC4483667

[B55] Ozturk O. Krehm M. Vouloumanos A. (2013). Sound symbolism in infancy: evidence for sound–shape cross-modal correspondences in 4-month-olds. *J. Exp. Child Psychol.* 114 173–186. 10.1016/j.jecp.2012.05.004 22960203

[B56] Paunović K. Stojanov V. Jakovljević B. Belojević G. (2014). Thoracic bioelectrical impedance assessment of the hemodynamic reactions to recorded road-traffic noise in young adults. *Environ. Res.* 129 52–58. 10.1016/j.envres.2014.01.001 24529003

[B57] Payne S. R. (2013). The production of a perceived restorativeness soundscape scale. *Appl. Acoust.* 74 255–263. 10.1016/j.apacoust.2011.11.005

[B58] Pheasant R. J. Fisher M. N. Watts G. R. Whitaker D. J. Horoshenkov K. V. (2010). The importance of auditory-visual interaction in the construction of ‘tranquil space’. *J. Environ. Psychol.* 30 501–509. 10.1016/j.jenvp.2010.03.006

[B59] Qin X. Kang J. Jin H. (2011). Subjective evaluation of acoustic environment of waiting areas in general hospitals. *Build. Sci.* 12 53–60.

[B60] Rashid M. Zimring C. (2008). A review of the empirical literature on the relationships between indoor environment and stress in health care and office settings: problems and prospects of sharing evidence. *Environ. Behav.* 40 151–190. 10.1177/0013916507311550

[B61] Recio A. Linares C. Banegas J. R. Díaz J. (2016). Road traffic noise effects on cardiovascular, respiratory, and metabolic health: an integrative model of biological mechanisms. *Environ. Res.* 146 359–370. 10.1016/j.envres.2015.12.036 26803214

[B62] Ren X. Kang J. (2015). Interactions between landscape elements and tranquility evaluation based on eye tracking experiments. *J. Acoust. Soc. Am.* 138 3019–3022. 10.1121/1.4934955 26627775

[B63] Shepley M. M. Baum M. Ginsberg R. Rostenberg B. (2009). Eco-effective design and evidence-based design: perceived synergy and conflict. *HERD* 2 56–70. 10.1177/193758670900200305 21165836

[B64] Spence C. (2011). Crossmodal correspondences: a tutorial review. *Atten. Percept. Psychophys.* 73 971–995. 10.3758/s13414-010-0073-7 21264748

[B65] Stamps A. E. III (2007). Evaluating spaciousness in static and dynamic media. *Des. Stud.* 28 535–557. 10.1016/j.destud.2007.01.001

[B66] Tajadura-Jiménez A. Larsson P. Väljamäe A. Västfjäll D. Kleiner M. (2010). When room size matters: acoustic influences on emotional responses to sounds. *Emotion* 10 416–422. 10.1037/a0018423 20515229

[B67] Thorgaard B. Henriksen B. B. Pedersbaek G. Thomsen I. (2004). Specially selected music in the cardiac laboratory—an important tool for improvement of the wellbeing of patients. *Eur. J. Cardiovasc. Nurs.* 3 21–26. 10.1016/j.ejcnurse.2003.10.001 15053885

[B68] Tobías A. Recio A. Díaz J. Linares C. (2015). Noise levels and cardiovascular mortality: a case-crossover analysis. *Eur. J. Prev. Cardiol.* 22 496–502. 10.1177/2047487314528108 24618478

[B69] Ulrich R. S. Simons R. F. Losito B. D. Fiorito E. Miles M. A. Zelson M. (1991). Stress recovery during exposure to natural and urban environments. *J. Environ. Psychol.* 11 201–230. 10.1016/s0272-4944(05)80184-7

[B70] Ulrich R. S. Zimring C. Zhu X. DuBose J. Seo H.-B. Choi Y.-S. (2008). A review of the research literature on evidence-based healthcare design. *HERD* 1 61–125. 10.1177/193758670800100306 21161908

[B71] Viollon S. Lavandier C. Drake C. (2002). Influence of visual setting on sound ratings in an urban environment. *Appl. Acoust.* 63 493–511. 10.1016/s0003-682x(01)00053-6

[B72] Von Lindern E. Hartig T. Lercher P. (2016). Traffic-related exposures, constrained restoration, and health in the residential context. *Health Place* 39 92–100. 10.1016/j.healthplace.2015.12.003 26995669

[B73] Ward R. D. Marsden P. H. (2003). Physiological responses to different WEB page designs. *Int. J. Hum. Comput. Stud.* 59 199–212. 10.1016/s1071-5819(03)00019-3

[B74] Watts G. Khan A. Pheasant R. (2016). Influence of soundscape and interior design on anxiety and perceived tranquillity of patients in a healthcare setting. *Appl. Acoust.* 104 135–141. 10.1016/j.apacoust.2015.11.007

[B75] Wu Y. Meng Q. Li L. Mu J. (2019). Interaction between sound and thermal influences on patient comfort in the hospitals of China’s Northern heating region. *Appl. Sci.* 9:5551 10.3390/app9245551

[B76] Xie H. Kang J. (2012a). Sound field of typical single-bed hospital wards. *Appl. Acoust.* 73 884–892. 10.1016/j.apacoust.2012.03.005

[B77] Xie H. Kang J. (2012b). The acoustic environment of intensive care wards based on long period nocturnal measurements. *Noise Health* 14 230–236. 10.4103/1463-1741.102960 23117538

[B78] Xie H. Kang J. Mills G. H. (2009). Clinical review: the impact of noise on patients’ sleep and the effectiveness of noise reduction strategies in intensive care units. *Crit. Care* 13:208. 10.1186/cc7154 19344486 PMC2689451

[B79] Xie H. Kang J. Mills G. H. (2013). Behavior observation of major noise sources in critical care wards. *J. Crit. Care* 28 e1105–e1109. 10.1016/j.jcrc.2013.06.006 23927941

[B80] Yin J. Zhu S. Macnaughton P. Allen J. G. Spengler J. D. (2018). Physiological and cognitive performance of exposure to biophilic indoor environment. *Build. Environ.* 132 255–262. 10.1016/j.buildenv.2018.01.006

[B81] Yost W. Zhong X. (2015). Localizing sound sources when the listener moves: vision required. *J. Acoust. Soc. Am.* 137 2373–2373. 10.1121/1.4920624

[B82] Yu C.-P. Lee H.-Y. Luo X.-Y. (2018). The effect of virtual reality forest and urban environments on physiological and psychological responses. *Urban For. Urban Green.* 35 106–114. 10.1016/j.ufug.2018.08.013

[B83] Zhang X. Lian Z. Ding Q. (2016). Investigation variance in human psychological responses to wooden indoor environments. *Build. Environ.* 109 58–67. 10.1016/j.buildenv.2016.09.014

[B84] Zijlstra E. Hagedoorn M. Krijnen W. P. Van der Schan P. Mobach M. P. (2017). Motion nature projection reduces patient\”s psycho-physiological anxiety during ct imaging. *J. Environ. Psychol.* 53 168–176. 10.1016/j.jenvp.2017.07.010

[B85] Zwicker E. Fastl H. (1999). Psychoacoustics. Facts and models. *Phys. Today* 54 64–65. 10.1063/1.1387599

